# Outcome and Cost Evaluation of Hip Fractures in Elderly Patients at a Tertiary Care Hospital in the Caribbean

**DOI:** 10.7759/cureus.74586

**Published:** 2024-11-27

**Authors:** Camille Sanderson-Jerome, Seetharaman Hariharan

**Affiliations:** 1 Anaesthesia and Intensive Care, Port of Spain General Hospital, Port of Spain, TTO; 2 Anaesthesia and Intensive Care, The University of the West Indies, St Augustine, TTO

**Keywords:** elderly hip fractures, healthcare outcomes, surgical costs, surgical hip repair, the caribbean region

## Abstract

Introduction

Hip fractures in the elderly are considered one of the most common types of orthopedic injuries, associated with increased morbidity and mortality. The incidence has been increasing over the years, and its management has also caused a significant economic burden for most countries worldwide. This study aimed to determine the outcomes and economic costs associated with hip fractures in elderly patients at a tertiary care institution in Trinidad and Tobago.

Methods

A chart review of patients aged > 65 years admitted with a diagnosis of hip fractures for one calendar year was conducted, followed by a prospective survey to determine their current status. Demographics and clinical data were collected. Outcomes measured included the time to surgery (lead time), complications by Clavien-Dindo score, hospital length of stay and mortality, and costs of hospitalization.

Results

Thirty patients who fulfilled the criteria for chart review and follow-up were studied. The age of patients ranged from 65 to 117 years with a mean of 82.0 ± 10.58 (standard deviation (SD)). The mean lead time from admission to surgery was 17.2 ± 14.2 (SD) days. The mean hospital length of stay was 24.4 ± 15.6 (SD) days. The mean cost of hospitalization was found to be US $10,256 per patient. Postoperative complications were seen in 57% of patients and non-surgical complications were more frequent than surgical complications. The longer the lead time to surgery, the longer the hospital length of stay (p<0.0001); the longer the length of stay, the higher the costs (p<0.0001). The hospital mortality was 27% and one-year mortality was 50%; the lead time to surgery did not impact hospital mortality.

Conclusion

Prolonged lead time until surgery for elderly hip fracture patients increased their hospital length of stay and significantly increased the costs of their management, adversely affecting their outcomes, which needs to be addressed at the administrative level.

## Introduction

Hip fractures have become one of the most common injuries in the elderly, and because of their pathophysiological effects, they are associated with high rates of morbidity and mortality [1]. In the year 2000, 1.6 million hip fractures were reported worldwide, of which approximately 620,000 occurred in Europe and another 250,000 in the United States. With increasing age, there is an exponential increase in the incidence of hip fractures. Due to advances in the field of medicine, the number of elderly people in the world has been steadily increasing, and the number of hip fractures in patients aged 50 years and above continues to rise. The projected number of hip fractures for the year 2050 is expected to be more than 6 million [2]. Hip fractures are associated with significantly high rates of morbidity and mortality, one-year reported mortality ranged from 10% to 37% [3]. Hip fractures are also linked to a significant decline in functional status, reduced ability to perform activities of independent daily living, and increased rates of admission into nursing homes. When the long-term outcomes of hip fractures were examined, more than half of the affected patients required assistance up to one year after their injury, half were unable to live on their own or perform basic activities of daily living independently and one-third were never able to walk without assistance [2,4,5]. The incidence and mortality of hip fractures must be studied in every setting as they provide essential information on population health metrics as well as improvements in healthcare [6].

Early corrective surgery for fractured hips has been linked to better functional outcomes, lesser pain, lower non-union rates, shorter hospital stays, and lesser postoperative complications [7]. However, the ideal timing of hip fracture surgery remains controversial. There is currently much debate in the literature about the potential effect of surgical lead time on the mortality rate of hip fracture patients. A study in 1995 by Zuckerman et al., reported that a surgical delay of over 48 hours was a positive predictor of one-year mortality in elderly patients who suffered hip fractures [8]. Subsequently, another paper, published in 2016 by Rosso et al., reported that corrective surgery should be performed under 48 hours to decrease the risk of mortality and that performing surgery between 48 and 72 hours after presentation to the hospital was not a satisfactory alternative [9]. A large, retrospective study by Pincus et al. concluded that surgical intervention within 24 hours of injury was linked to a significant reduction in 30-day mortality, fewer complications, and fewer adverse outcomes at 30 days [10]. There are also a few studies in which experts advocate performing ultra-early surgery within 12 hours of admission [11]. In contrast, there are some studies that found no meaningful association between longer surgical delays and mortality [12-14]. Experts against early corrective surgery have suggested that a period of operative delay can acceptably be utilized for optimizing or stabilizing elderly patients since these patients are usually frail and present with multiple comorbidities and polypharmacy [7]. The current guidelines from the National Institute for Health and Care Excellence (NICE), UK, recommend that adults who have suffered hip fractures undergo corrective surgery on a planned operative trauma list within 48 hours of their admission to the hospital [15].

Careful estimation of the total healthcare costs incurred from hip fractures is necessary to carry out analyses by health economic divisions, which can then guide policy decisions related to the distribution and allocation of health resources [16]. A large retrospective study by Kempenaers et al. showed that overall healthcare costs rose steadily with increasing surgical delay for hip fractures [7].

In our setting, on average, patients admitted to the hospital after sustaining hip fractures undergo surgery within one month of their admission. This delay may be due to the unavailability of implants, inadequate operating theatre time, or patient comorbidities requiring optimization. Despite existing guidelines, there is no dedicated trauma list on which these patients can be operated on in an expeditious manner. This contributes to the surgical delay and possibly increases mortality and healthcare costs. However, to our knowledge, there have been no published studies regarding the delays to hip fracture surgery and the associated increase in costs in such patients from the Caribbean.

With this background, the present study attempted to evaluate the outcome of hip fractures in elderly patients at a teaching tertiary care hospital, the factors influencing the outcome, as well as the costs of treating such patients in a Caribbean teaching hospital.

## Materials and methods

This was a retrospective chart review along with a prospective follow-up study to determine the outcomes and costs of managing elderly patients with hip fractures. Approval of the Ethics Committee of the Regional Health Authority was obtained for the study. Initially, the particulars of the patients were obtained from the orthopedic wards’ admissions books and then the patient files were located in the medical records department. Personal identifying information of the patients was not recorded and the patient data were codified. The retrospective chart review had a waiver of consent, while for the prospective follow-up, informed consent was obtained before administering the questionnaire to the patients or their relatives.

The study included elderly patients (aged 65 years and above) admitted with a diagnosis of hip fracture during the period from January 1st to December 31st, 2017. A total of 120 patients were admitted with a diagnosis of hip fractures during this period, which included patients of all ages. Of the 120 patients, only 74 patient files could be located. Twenty-three (23) of the located files were found to have other diagnoses after initial assessment by the orthopedic teams, 22 patient files were missing the portions that dealt with the admission for the management of their hip fractures, and two patient files were repeat procedures.

Patients less than age 65 years, patients for revision hip surgery, and those who were diagnosed with avascular necrosis of the head of the femur were excluded from the study.

Demographic data collected included age and gender. Clinical data included preoperative information such as American Society of Anesthesiologists (ASA) grade and comorbidities, the dates and times when the patient sustained the fracture, the date when they were brought to the Accident & Emergency (A&E) Department for admission, length of stay at the A&E Department, type of fracture, length of stay in orthopedic wards, type of anaesthesia, type of surgery, duration of surgery, type of implant inserted, postoperative complications quantified by Clavien-Dindo score (which ranges from 1 to 5, 1 = mild complications e.g., wound infections; 2 = complications requiring pharmacological interventions; 3 = those requiring surgical interventions; 4 = organ failure with ICU type of intervention, and 5 = patient demise), postoperative length of stay, and overall hospital outcome.

Patients or their next of kin were then contacted to determine their outcome after a year with the help of a structured questionnaire. Patients or relatives were contacted using a designated cellular phone and after obtaining consent, the questionnaires were filled out.

The costs of the overall hospitalization were calculated by collecting data on a daily basis from the day of admission until the day of discharge or death, including investigations done pre- and postoperatively, the drugs used perioperatively, the implants used, and the activity-based costing for procedures undertaken. The overall costs were computed using the current cost of materials, such as surgical implants and pharmaceuticals, obtained from the currently available National Costing of Health Services published by the Health Economics Unit, and the Ministry of Health Pharmaceutical Price List. The costs of various surgical implants were obtained from the companies that supply instruments and implants.

Descriptive as well as inferential statistical analyses were done using ​Statistical Package for Social Sciences (SPSS) - version 21 (IBM Corp., Armonk, USA). Appropriate inferential tests such as t-tests, Pearson Chi-square tests, independent Mann-Whitney and Kruskal-Wallis tests, and Pearson Correlations were performed for comparisons. Receiver Operating Characteristic curve analysis was used for the discriminant function of factors affecting hospital mortality. The statistical significance was fixed at the level of p<0.05.

## Results

Data from thirty (30) patient files were collected for this study. However, since data for each patient were collected from admission to discharge on a daily basis for costing purposes, the total data points amounted to 1020.

The age of the patients ranged from 65 to 117 years with a mean of 82.0 (±10.6 SD) years. The cohort consisted of 13 (43.3%) male and 17 (56.7%) female patients. Patients were categorized by ASA grade, which revealed that most patients (16 (53.3%)) belonged to the ASA III category.

Preoperative comorbidities that required optimization included hypertension, diabetes mellitus, anaemia, pneumonia, electrolyte imbalance, chronic obstructive pulmonary disease (COPD) and decubitus ulcer. The number of comorbidities ranged from 0 to 5 in these patients on admission to the hospital. The most common comorbidity was hypertension, which was seen in 17 (56.7%) patients, followed by anaemia in 10 (33.3%) and diabetes mellitus in eight (26.7%) patients. Table 1 shows the demographic data of the patients.

**Table 1:** Demographic data ASA= American Society of Anesthesiologists Physical Status; SD = Standard Deviation

Variable	Distribution					
Age (y) *Range (Mean±SD)*	65-117 (82 ± 10.6)					
Gender n (%)	Male			Female		
	13 (43.3)			17 (56.7)		
ASA Category n (%)	I		II	III	IV	
	1 (3.3)		10 (33.3)	16 (53.4)	3 (10.0)	
Number of comorbidities n (%)	0	1	2	3	4	5
	3 (10.0)	9 (30.0)	6 (20.0)	8 (26.6)	2 (6.7)	2 (6.7)

Table 2 shows the type of fractures according to the sex of the patients.

**Table 2:** Type of fractures in males and females

Sex	Fracture type				
	Intertrochanteric	Subcapital	Transcervical	Basicervical	Subtrochanteric
Female	10	4	1	1	1
Male	4	6	1	1	1
Total n (%)	14 (46.6)	10 (33.3)	2 (6.7)	2 (6.7)	2 (6.7)

The most common type of fracture seen was intertrochanteric 14 (46.6%) followed by sub-capital 10 (33.3%) type. Two patients each (6.7% each) sustained basicervical, transcervical, and subtrochanteric fractures. Intertrochanteric fractures affected more female patients than males. All other fracture types showed comparable distribution among male and female patients. There was no statistically significant difference in the type of hip fracture sustained between males and females (Pearson Chi-square value 2.53; df: 4; p = 0.64).

The type of surgery performed and implant used varied based on fracture type. Ten patients (33.3%) of patients were treated with Dynamic Hip Screw placements, and another 10 (33.3%) patients underwent Bipolar Hemiarthroplasties. Conservative management was the chosen option for eight (27%) patients, while one patient each (3.3% each) underwent Total Hip Replacement and Intramedullary Nail Placement. Of the patients who underwent corrective surgery for their hip fractures, 14 (64%) received a regional anaesthetic technique while eight (36%) received a general anaesthetic. There was no statistically significant difference in the 30-day mortality of patients with respect to the anaesthetic technique used (Pearson Chi-square value 0.049; df: 2; p=0.976).

**Figure 1:**
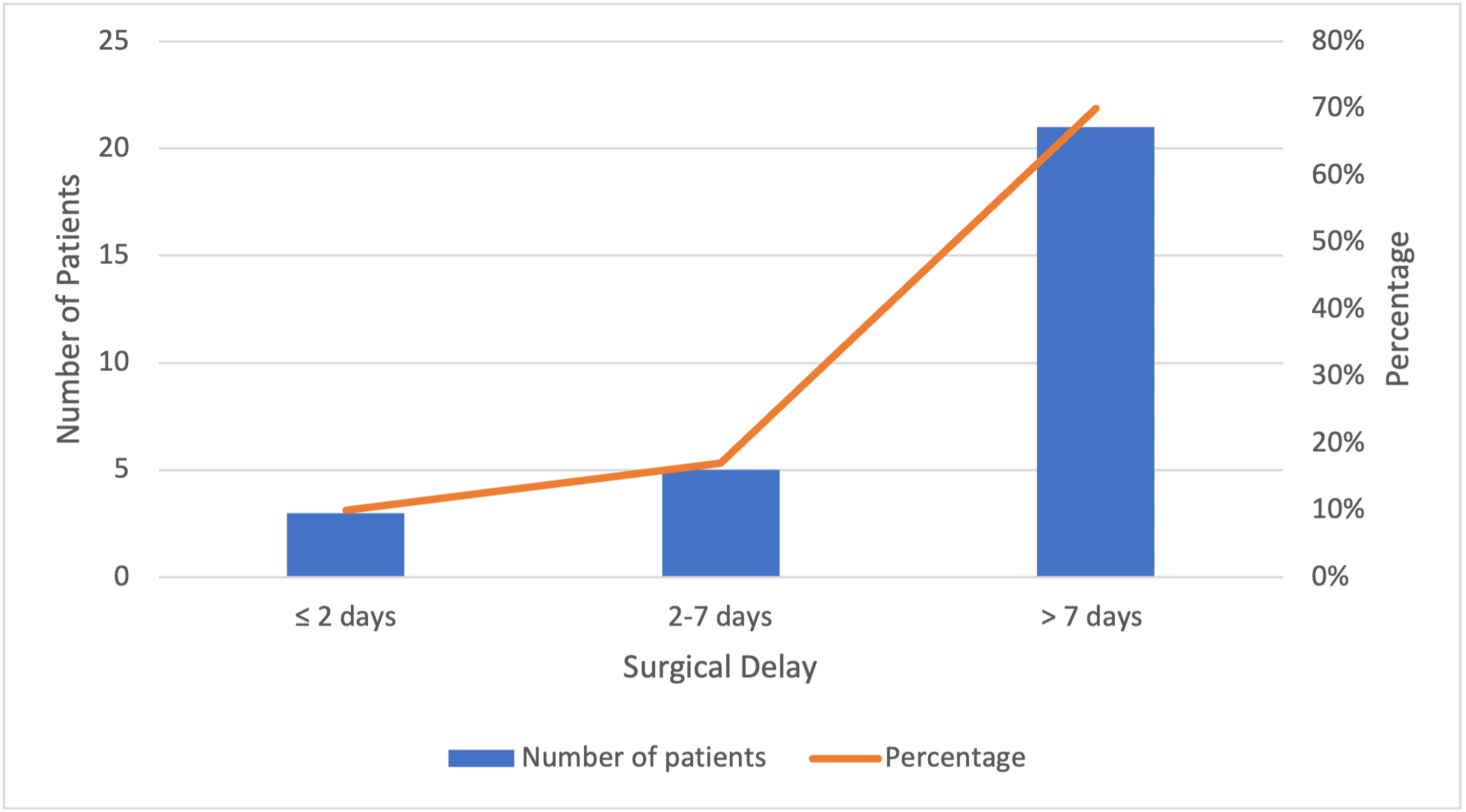
Timing of operation

Nineteen (63%) patients presented to the Accident and Emergency (A&E) department within 24 hours of sustaining their fracture, while three (10%) presented within 48 hours. Four (13%) patients presented after 48 hours but under 96 hours post injury, while another four (13%) patients presented more than 144 hours after their fracture. On average, the time between patient admission to the orthopaedic ward from the A&E was 4.2 hours. The mean time between patient fracture and surgery (fracture delay) was 18.9 days while the mean time between admission and surgery (surgical delay) was 17.2 days. The proportions of patients operated on within 48 hours, a week, and more than a week are shown in Figure 1.

**Figure 2:**
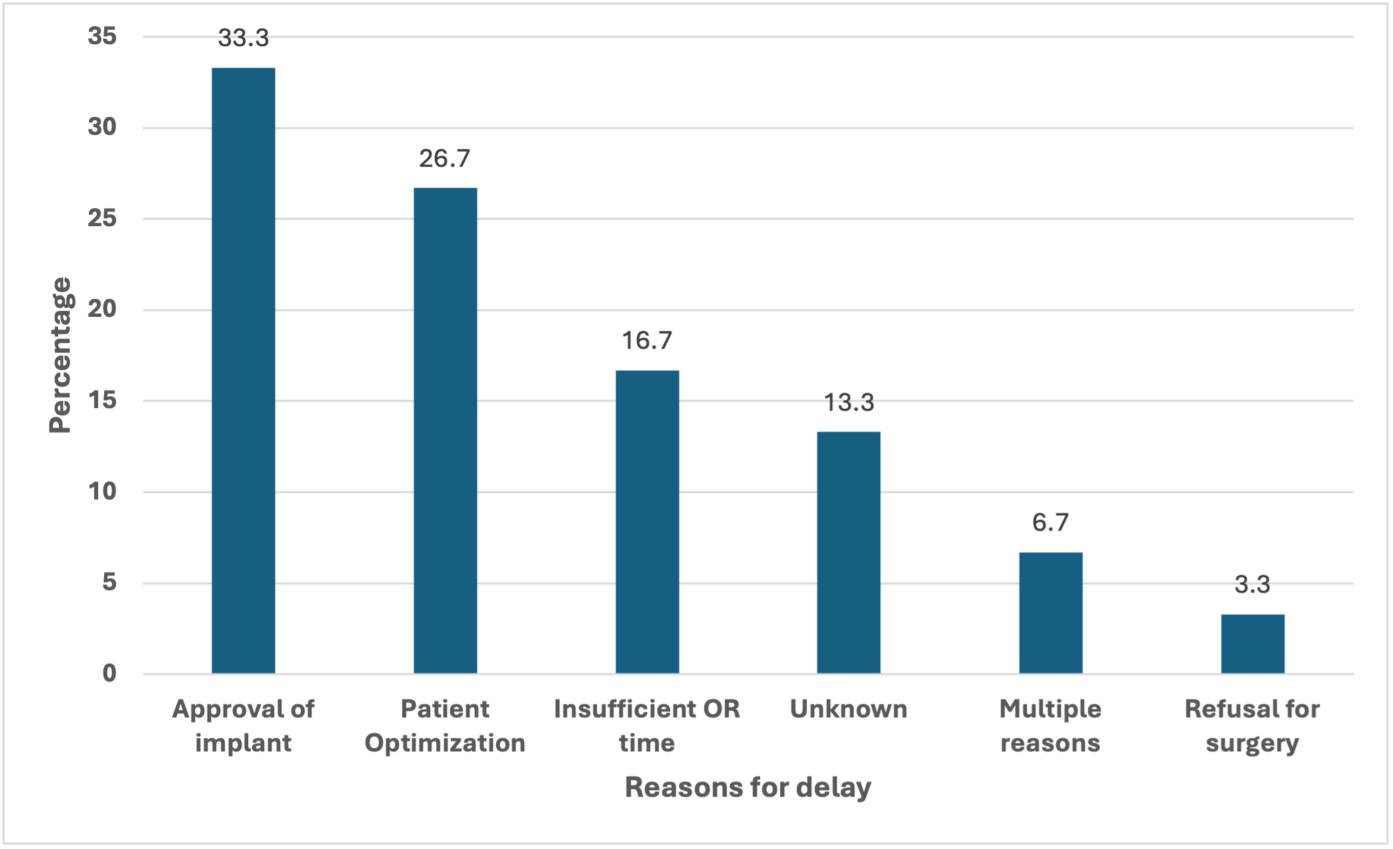
Reasons for surgical delay OR = Operating Room

The various reasons for surgical delay are shown in Figure 2.

The reasons for the surgical delay included awaiting approval for the surgical implant, insufficient operating time, time for patient optimization, and patient refusal. Two patients had multiple reasons contributing to their surgical delay time. The most common reason for surgical delay was lack of approval for purchasing surgical implants - 10 (33%). Patient optimization was the second most common reason for surgical delay in eight (27%) patients. The third most common reason for surgical delay was insufficient operating time, which affected five (17%) patients. Patients are usually scheduled to have their surgeries on elective orthopaedic operating lists, which consist of 8 hours of operating time two to three days per week. 

The patients stayed ranging from 1 to 56 days in the hospital with an average length of stay in the hospital of 24.4 days (± 15.6 SD). For most patients, the major portion of the hospital stay was predominantly the delay due to waiting for surgery. The surgical delay time correlated with the length of hospital stay (Pearson correlation coefficient 0.873; p<0.0001).

**Figure 3:**
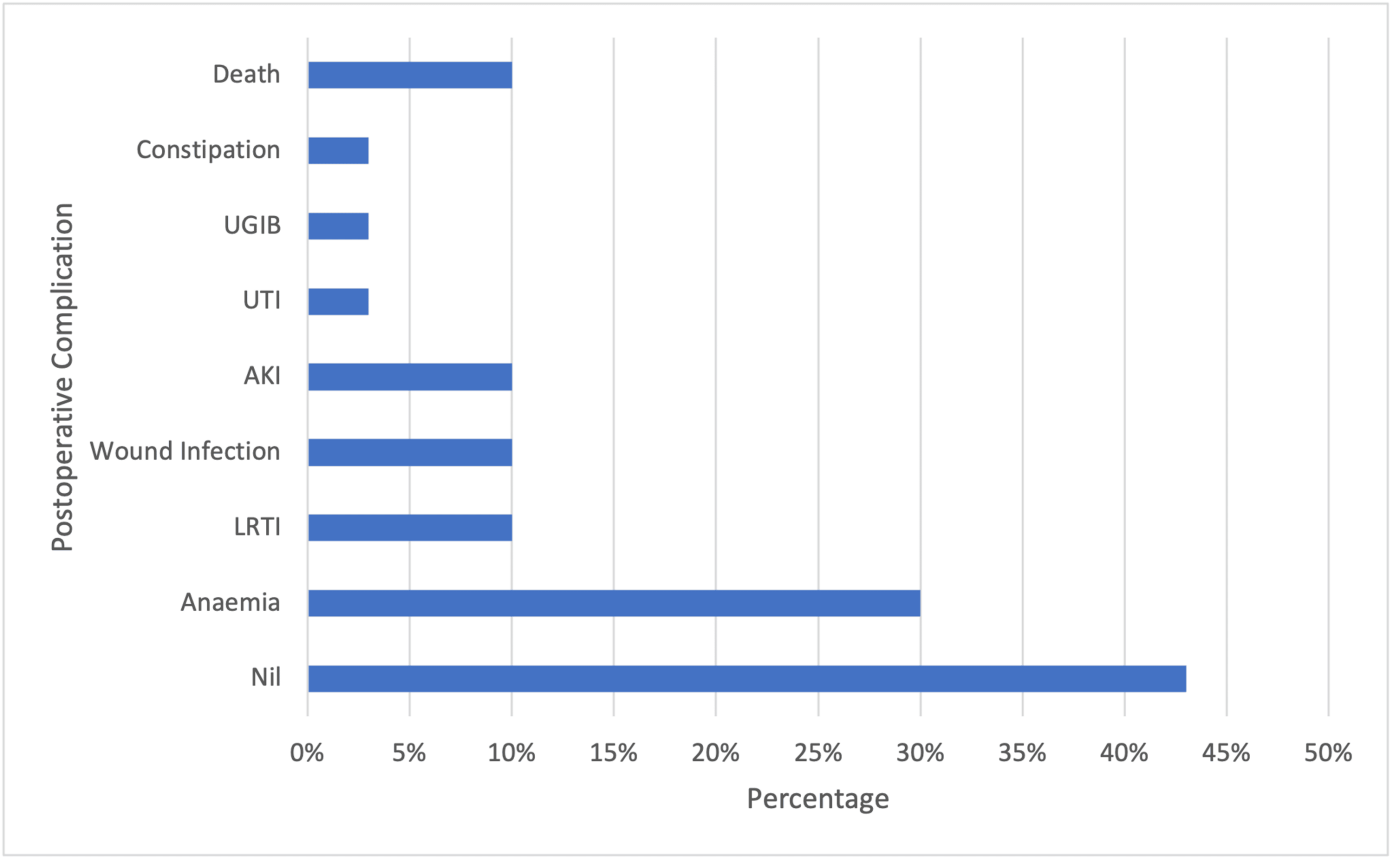
Postoperative complications UGIB = Upper gastrointestinal bleed; UTI = Urinary Tract Infection; AKI = Acute Kidney Injury; LRTI = Lower Respiratory Tract Infection

Patients who had a postoperative length of stay of more than seven (7) days were those in whom preoperative morbidity and/or postoperative complications were present. Postoperative complications were seen in 17 (56.7%) patients. These complications were mostly non-surgical and included anaemia and lower respiratory tract infections. Wound infection was the only surgical complication reported and this was found in three (10%) patients. The mean Clavien-Dindo score for these patients was found to be 1.4. Postoperative complications are shown in Figure 3.

The total costs ranged from US $541.17 to US $21.948.11, with the mean cost of hospital admission for patients with hip fractures being US $10, 253.90. The main contributors to cost were the length of stay and the number of investigations performed, which depended heavily on the number of comorbidities and the presence of postoperative complications. The type of surgical implant, surgical duration, and medications were other contributors to cost.

**Figure 4:**
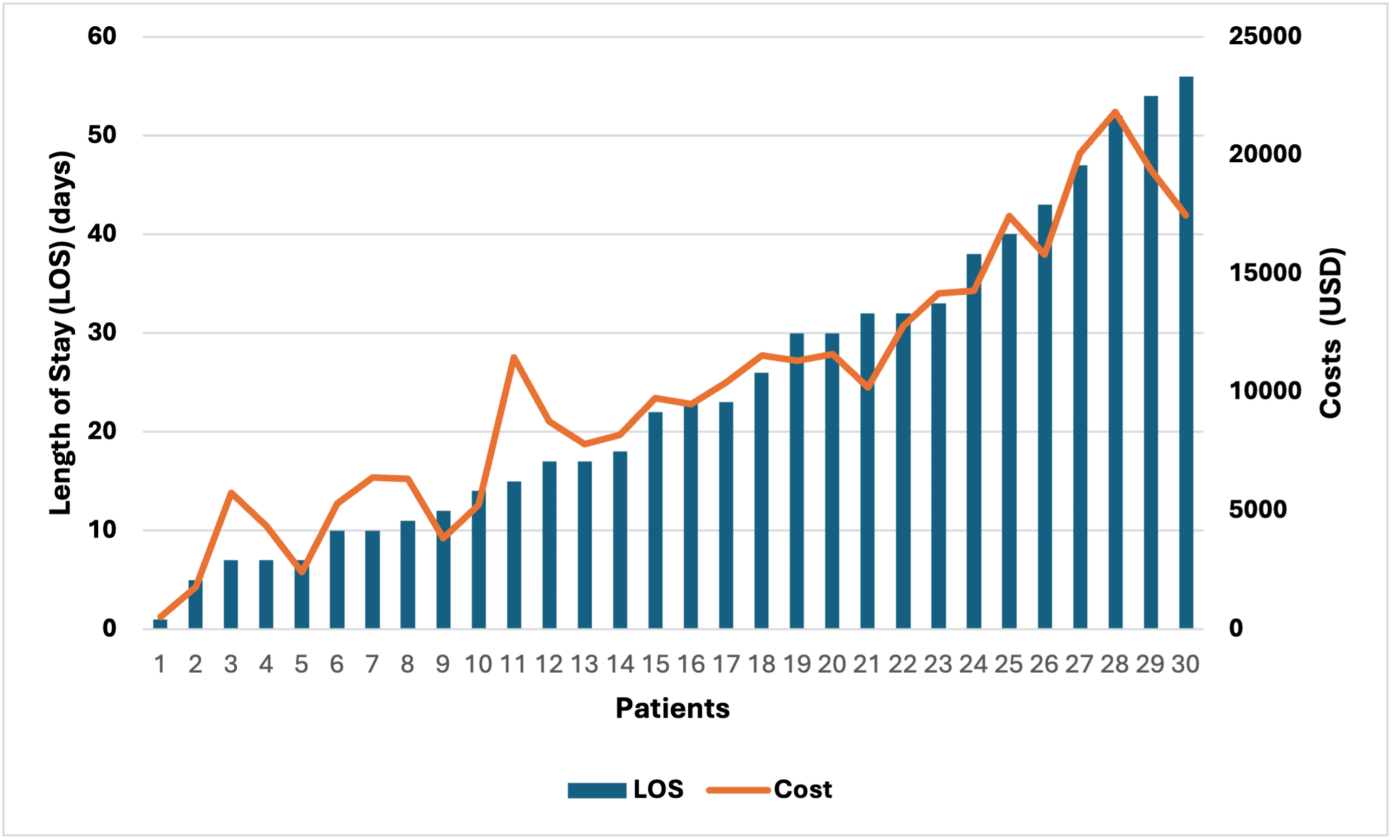
Length of stay and costs

The total cost was found to be directly proportional to the length of stay (Pearson correlation coefficient 0.956; p<0.0001). The costs were also proportional to the number of investigations required. Figure 4 shows the relationship between the length of stay and the total average cost.

**Figure 5:**
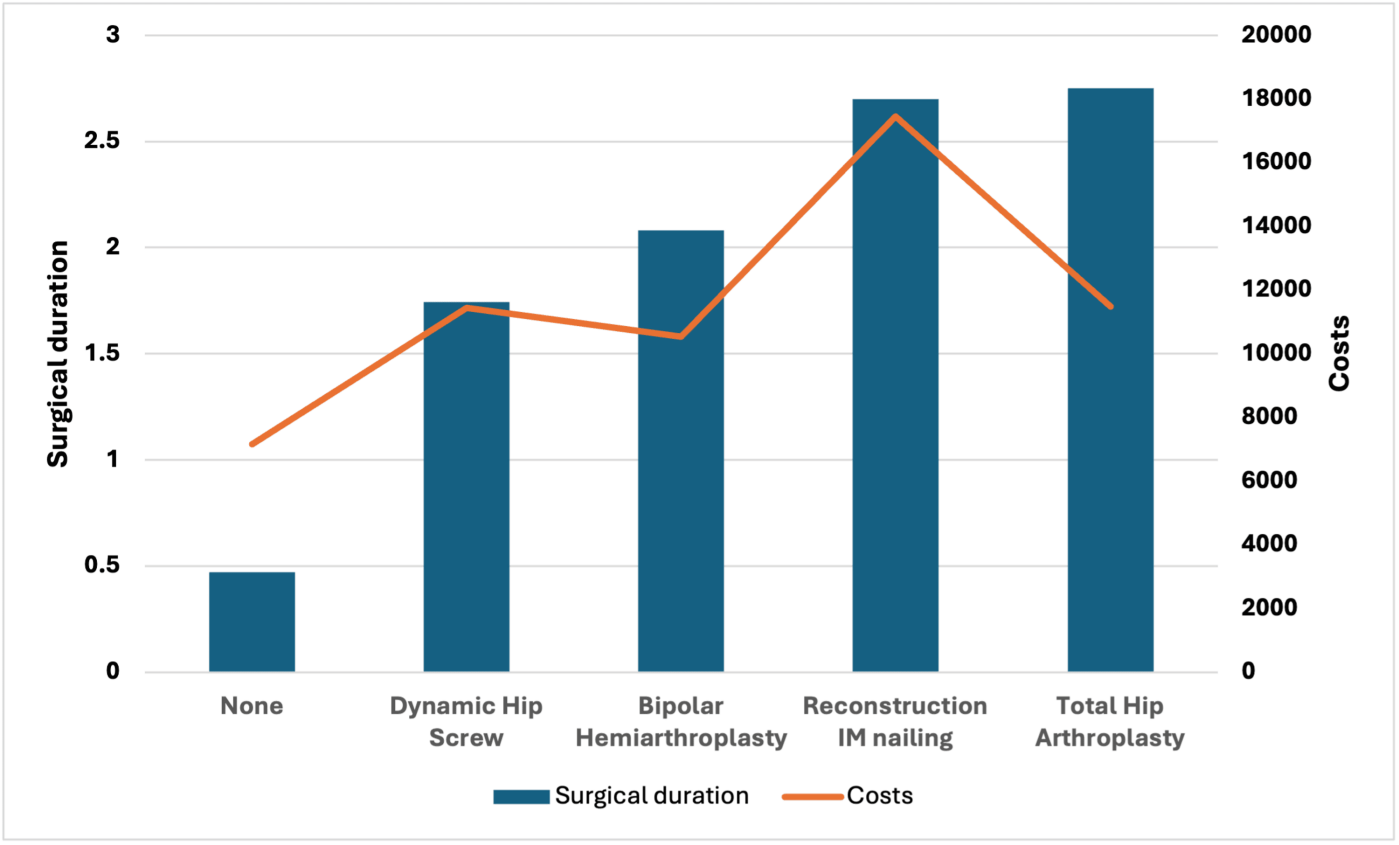
Surgical procedure, duration and costs IM = intramedullary

The surgical cost varied with the type of surgery and the choice of implant used. In addition, surgical cost was also affected by the duration of the surgical procedure. The average surgical duration was 1.46 hours (± 0.99 SD) with an average surgical cost of US $14,744.63. The total estimated cost for the studied cohort was US $306,800. Figure 5 shows the effects that the type of surgery and surgical duration had on the average surgical cost.

The insertion of a Dynamic Hip Screw was associated with the shortest surgical duration and lowest surgical cost, whereas a Total Hip Arthroplasty was associated with the longest surgical duration and Reconstruction IM Mailing was associated with the highest surgical cost.

Eight patients died within a month of their fracture; hence the 30-day mortality was 26.7%. An additional seven patients died within one year following their hip fracture, resulting in a one-year mortality of 50%. Pearson Chi-square test showed no statistically significant difference within the sexes with respect to one-year mortality (Chi-square value 0.179; df: 2; p = 0.914). Two patients out of eight in the conservative management group, four of 14 in the regional anaesthetic group, and two of eight in the general anaesthetic group died, and there was no statistically significant difference in the 30-day mortality of patients with respect to the anaesthetic technique (Pearson Chi-square value 0.049; df: 2; p=0.976)

Table 3 shows the comparison of age, length of stay, surgical duration, Clavien-Dindo score, and total costs between survivors and non-survivors during their hospital admission. Because of the data distribution, a non-parametric test (Mann-Whitney U test) was used for comparison.

**Table 3:** Comparison of variables between survivors and non-survivors ^1^p-value by Mann-Whitney U test; ^2^p-value by Chi-square test; LOS = length of stay; USD = United States Dollar

Variable	Survivors (n=22)	Non-survivors (n=8)	Significance
Age (Mean ± SD)	79.8 ± 10.9	87.9 ± 6.9	p=0.010^1^
Gender (n) Male	10	3	p=0.515^2^
Female	12	5	
Surgical delay (days) (Mean ± SD)	18.9 ± 14.6	13.1 ± 12.4	p=0.298^1^
Hospital LOS (days) (Mean ± SD)	24.8 ± 14.2	23.3 ± 20.1	p=0.629^1^
Clavien Dindo Score (Mean ± SD)	0.64 ± 1.1	2.63 ± 2.2	p=0.040^1^
Total Costs (USD) (Mean ± SD)	10,132.97 ± 4469.89	10,379.61 ± 8366.40	p=0.801^1^

**Figure 6:**
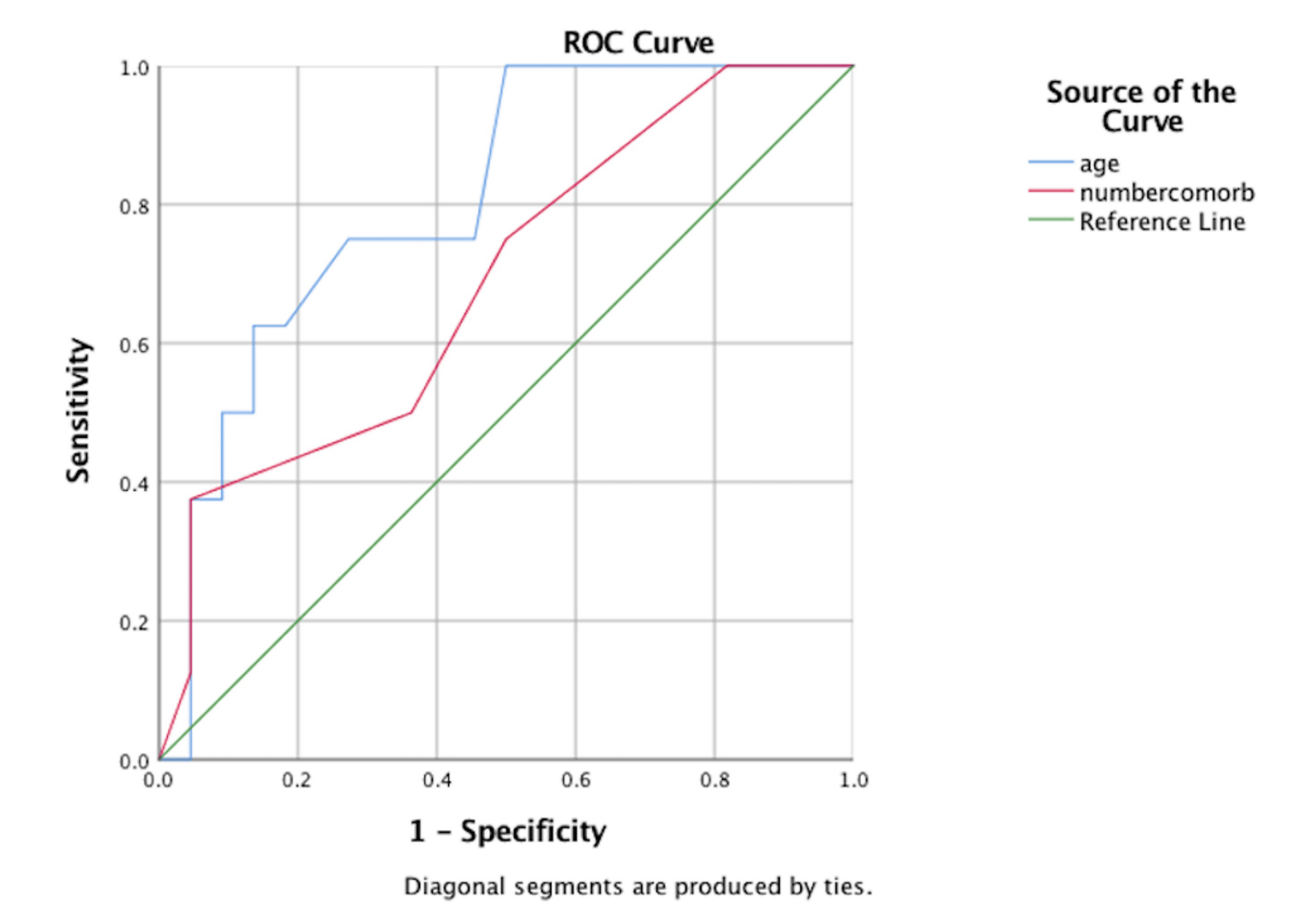
Receiver operating characteristic (ROC) curves for age and the number of comorbidities numbercomorb = number of comorbidities

Receiver operating characteristic (ROC) analysis showed a reasonably good discriminant function for age (area under the curve (AUC): 0.807; 95% confidence intervals: 0.642, 0.972); p=0.011) with respect to 30-day mortality. The number of comorbidities had a reasonable AUC of 0.688; however, confidence intervals were 0.473, 0.902; and the p-value was 0.12. Figure 6 shows the ROC curve for these two factors.

Also, there was no statistically significant influence of surgical delay or the overall length of stay on 30-day mortality (AUC 0.44 and 0.37; p=0.62 and 0.29, respectively). Kruskal-Wallis independent samples analyses showed no statistically significant difference between the type of fracture and the surgical delay (p=0.277) as well as the total costs (p=0.237).

## Discussion

The present study could quantify the significant burden of hip fractures in the elderly on the country’s healthcare system and economy. In addition, the present study reported many characteristics of a cohort from the Caribbean that were comparable to most studies on hip fractures from other regions. However, the most notable difference in the present study was the higher average length of stay when compared with other studies. Several studies reported an average in-hospital stay in the range of 13 to 20 days, whereas the average length of hospital stay in the present study was 24.4 days. This prolonged length of stay was primarily due to a longer time taken for surgical intervention; very few hip fracture patients had surgery within 48 hours or less. Instead, the average surgical delay was found to be in excess of 15 days with the main reasons being patient optimization, lack of financial approval from authorities for surgical implants and inadequate operating time. A previous study found that earlier surgery (within 72 hours) was associated with reduced postoperative complications and mortality [17]. A systematic review and meta-analysis concluded that early corrective surgery for hip fractures was associated with reduced mortality rates and fewer complications in the postoperative period [18]. One study advocated lead-time to optimize patients’ comorbid illnesses, although it could not establish that preoptimization improved the overall outcome of hip fracture patients [17]. Current guidelines suggest that surgery for patients with hip fractures should be performed within 24 hours of admission as much as possible since reduced surgical lead-time has been linked to improved function postoperatively, shorter duration of pain, reduced hospital stay and reduced rates of complications and mortality [19]. NICE guidelines recommend having a dedicated trauma list so that these patients can have their surgery in an expeditious manner. However, currently, no such trauma list exists in the study hospital and these patients must be done during the limited elective orthopaedic operating time.

The one-year mortality of elderly patients with hip fractures has been reported to range between 14% and 36% [3, 20]. In the present study, the one-year mortality was found to be 50%, and the overall lead time to surgery was higher. This higher mortality rate with prolonged surgical delays has been corroborated by the findings of Vidán et al., where higher rates of mortality and medical complications were associated with surgical delays of more than 120 hours [21]. The meta-analysis by Simunovic et al. also found that earlier surgery decreased the risk of one-year mortality by almost 50% [18]. The correlation between surgical delay and mortality could not be established in our study most likely because of the small sample size. The sex of the patient and the type of fracture were not significant factors to be associated with high mortality in the present study. However, ROC analysis did show that the age of the patient and the number of comorbidities may be the factors influencing mortality.

In the current study, postoperative complications were seen in 57% of patients with 10% experiencing surgical complications. Comparable studies on hip fractures showed postoperative complication rates of 12.5% to 40% [22]. The main surgical complication seen in this study was wound infection whereas the main non-surgical complications seen were anaemia (30%) and lower respiratory tract infection. Anaemia accounted for the majority of non-surgical complications in most hip fracture studies, with some authors reporting rates as high as 86%. A Spanish meta-analysis reported rates of postoperative anaemia ranging between 24% to 44% and a retrospective cohort study conducted by Saul et al. found a rate of 39.8% [23]. There was no difference seen between the type of surgery performed and the incidence of surgical or non-surgical complications, which is consistent with the findings of other studies.

Hip fractures are costly as they demand substantial resources from a country’s healthcare system. The direct cost of hip fractures according to the model by Hernlund et al. consisted of hospitalization costs, rehabilitation costs, and nursing costs [24]. The average cost per patient of acute hospital care for hip fractures in the present study was US $10,256.79, which included in-hospital stay, radiological and blood investigations, corrective surgery, physiotherapy, and medications. When compared to the cost of acute hospital care for hip fractures in various countries, our cost per patient was lower than that of the US (US $33,000), UK (£12,163) and Finland (€14,410) but more than France (€8048 to €8727) and Singapore (US $8224.76) [25-27]. The factors that were found to be associated with higher costs included length of stay and number of comorbidities, but not the type of fracture. The hospitalization costs increased by US $295 per day, which excluded any investigations and medications required. The presence of comorbid illnesses also influenced the total costs as they significantly increased the cost of care. One study recommended focusing management on those comorbidities that have the greatest impact on hospitalization costs [25]. The present study showed a general increase in hospital costs with an increasing number of comorbidities.

There were several limitations in this study. Firstly, this study was conducted retrospectively and currently, there are no electronic patient records at the study hospital. Incomplete files accounted for approximately 18% of the requested patient files. Secondly, the total number of patients was 30, which may be considered a small sample size; however, since they were tracked on a daily basis from admission to discharge, the total data points were 1020. Another possible limitation is the non-inclusion of the rehabilitation costs. There was no documentation of rehabilitation sessions in patients’ hospital files; therefore, this component of the direct cost could not be included in this study.

## Conclusions

Nevertheless, in summary, this study found that there was an inordinate surgical delay in managing hip fractures in the elderly that far exceeds that of current guidelines around the world. The one-year mortality was also higher than most comparable hip fracture outcome studies.

The factors associated with adverse outcomes were both non-correctable and correctable. The non-correctable factors included the age of the patients and the number of comorbidities, while the correctable factors included the surgical delay time and hospital length of stay. Although the non-correctable factors cannot be addressed directly, steps such as preoperative optimization can be undertaken to partially address them. The surgical delay time and length of stay can be addressed directly. In this season of economic scarcity, this research found that the direct hospitalization costs for the management of hip fractures in an elderly cohort are US $306,800.This has highlighted not only the economic burden but also the importance of following the current guidelines for the perioperative management of hip fractures in elderly patients.
